# Performance of Clinical Algorithms for Smear-Negative Tuberculosis in HIV-Infected Persons in Ho Chi Minh City, Vietnam

**DOI:** 10.1155/2012/360852

**Published:** 2012-11-25

**Authors:** Duc T. M. Nguyen, Hung Q. Nguyen, R. Palmer Beasley, Charles E. Ford, Lu-Yu Hwang, Edward A. Graviss

**Affiliations:** ^1^Department of Pediatrics, University of British Columbia and BC Children's Hospital, 4480 Oak Street, A4-198 AD, Vancouver, BC, Canada V6H 3V4; ^2^Department E (HIV Infection), Hospital for Tropical Diseases (HTD), Ho Chi Minh City, Vietnam; ^3^Division of Epidemiology, Human Genetics & Environmental Sciences, University of Texas School of Public Health (UTSPH), Houston, TX 77030, USA; ^4^Division of Biostatistics, University of Texas School of Public Health (UTSPH), Houston, TX 77030, USA; ^5^Department of Pathology and Genomic Medicine, The Methodist Hospital Research Institute (THMRI), Houston, TX 77030, USA

## Abstract

*Background*. Tuberculosis (TB) disease diagnosis in Vietnam relies on symptom screening, chest radiography (CXR), and acid fast bacilli (AFB) sputum smear which have a poor sensitivity in HIV patients. We evaluated the performance of clinical algorithms in screening and diagnosing AFB smear-negative TB in HIV patients. *Methods*. We enrolled 399 HIV-positive patients seeking care at a HIV clinic in Ho Chi Minh City (HCMC), Vietnam. Participants' demographics, medical history, common TB symptoms, CXR, and laboratory tests were collected. *Results*. Of 399 HIV patients, 390 had initial AFB-negative smears and 22/390 patients had positive cultures. Symptom screening missed 54% (12/22) of smear-negative pulmonary TB (PTB) cases. Multivariate analysis found CD4+ cell level and CXR were significant PTB predictors. An algorithm combining four TB symptoms and TST presented a high sensitivity (100%), but poorly specific (24%) diagnostic performance for smear-negative PTB. *Conclusion*. Up to 54% of PTB cases in the HIV-infected population may be missed in the routine screening and diagnostic procedures used in Vietnam. Symptom screening was a poor overall diagnostic measure in detecting smear-negative TB in HIV patients. Our study results suggest that routine sputum cultures should be implemented to achieve a more accurate diagnosis of TB in HIV patients.

## 1. Introduction

HIV-infected patients with tuberculosis (TB) coinfection may present with atypical manifestations of pulmonary TB (PTB) and a higher rate of negative sputum smears for acid fast bacilli (AFB) [1–3]. Though being less infectious than AFB smear-positive PTB, AFB smear-negative PTB is still a potentially important source of TB transmission and predicts a worse prognosis for HIV-infected patients [4–6]. Therefore, early and accurate diagnosis of sputum smear-negative PTB for HIV-infected patients is urgently important for not only improving the patient's life expectancy, but also preventing disease transmission to the general population. 

The diagnosis of TB disease in developing countries like Vietnam relies mainly on symptom screening, chest radiography (CXR), and AFB sputum smear, which have a poor sensitivity in HIV-positive patients [3, 7]. Sputum culture is not routinely available for smear-negative patients, especially at district-level public health institutions [7]. Moreover, current data on the performance of clinical, radiographic, and laboratory characteristics in predicting AFB smear-negative PTB for HIV-infected patients are still limited and inconsistent [6, 8]. 

This reasoning led us to evaluate the diagnostic performance of available clinical, radiographic, and laboratory factors in diagnosing AFB smear-negative PTB for HIV-infected patients at a district-level HIV clinic in Ho Chi Minh City (HCMC), Vietnam. Data obtained from this study will help develop a standard algorithm for AFB smear-negative PTB in areas where both HIV and TB are prevalent and provide the basis for future studies on more accurate and rapid tools for TB diagnosis. 

## 2. Material and Methods

### 2.1. Enrollment

This study was conducted at the An Hoa Clinic, a neighborhood HIV clinic located in District 6, HCMC, Vietnam. An Hoa Clinic is where approximately 65% of District 6's HIV patients come for screening of opportunistic infections and receiving antiretroviral therapy (ART) [9, 10]. 

To our knowledge, this was the first study to investigate the prevalence of AFB smear-negative PTB in HIV-infected patients in Vietnam. As there was no preliminary published data for calculating power or sample size, we used a convenient sample size calculation for this pilot study. In this cross-sectional study, 399 HIV-infected subjects were consecutively enrolled from August 2009 through June 2010. Inclusion criteria included: (a) District 6 residents, (b) age ≥ 15 years, (c) had not been treated for TB during the past year, and (d) laboratory-confirmed HIV infection.

### 2.2. Data Collection

This study was part of the “Tuberculosis in HIV-infected individuals in Ho Chi Minh City, Vietnam” project and the detailed methodology has been described elsewhere [11]. Briefly, written informed consent was obtained for all study participants before subjects were interviewed. A standardized data collection form was used to gather information on participants' demographics, medical history, TB symptoms, and laboratory results.

After interview, participants were referred to the Pham Ngoc Thach Hospital for Tuberculosis and Lung Diseases (PNT) in District 5, HCMC for chest radiography (CXR), tuberculin skin test (TST), AFB sputum smears and cultures, and CD4+ cell count.

CXR was conventionally performed on posterior-anterior view (PA) using large films and read by a trained blinded radiologist. The radiologist reviewed radiographs for TB-specific presentations such as cavitations, military tuberculosis, consolidation, infiltrates, nodules, fibrosis, pleuritis, and adenopathy. CXRs with TB-specific presentations were classified as abnormal CXR suggestive for TB disease (TB-CXR). 

TST was placed on the volar surface of participants' forearm by intradermally injecting 5 tuberculin units of purified protein derivative (PPD-Hanoi, Vietnam) [12]. Results were read after 48–72 hours of placement. An induration of at least 5 mm was classified as positive [13]. CD4+ cell level in participants' plasma was recorded in cell/mm^3^.

Two sputum specimens were collected for each participant as per World Health Organization (WHO)'s guidelines [14], one at home and the other at the PNT Hospital in the early morning. Sputum specimens were decontaminated by NaOH-NALC 2% solution before being aliquoted for smear examination and culture [15]. Direct sputum microscopic examination was performed for acid fast bacilli (AFB) using the Ziehl-Neelsen method as per the Vietnamese National TB Program Guidelines [7]. Sputum smear results were categorized as smear-negative, when both smears were negative for AFB, or smear-positive, when at least one smear was positive for AFB. Sputum cultures were also performed on the Löwenstein-Jensen medium for *Mycobacterium tuberculosis *(MTB). We applied one sputum culture per patient as per the routine procedure at the PNT hospital. The cultures were consistently performed on the second specimens which were collected at the hospital. MTB was differentiated from mycobacteria other than tuberculosis (MOTT) by the niacin accumulation test. A subject was classified as a pulmonary TB (PTB) case when he/she had a sputum culture positive for MTB [16]. 

MTB isolates were assessed for the susceptibility to rifampin, isoniazid, streptomycin, and ethambutol by the Canetti-Grosset proportion method [17]. Resistant TB was defined as isolate resistant to at least one anti-TB drug. MDR-TB was defined as an isolate resistant to rifampin and isoniazid [16]. 

### 2.3. Ethics

This study was approved by the Institutional Review Boards (IRBs) of The Methodist Hospital Research Institute (TMHRI) and HCMC Provincial AIDS Committee (PAC).

### 2.4. Data Analysis

As the main objective of this study was to evaluate the performance of potential predictors in diagnosing smear-negative, culture-positive PTB, the data set used in this analysis included only HIV-infected patients having sputum smears negative for AFB. Univariate odds ratios (OR) with 95% confidence intervals (CI) were calculated to evaluate the possible association between smear-negative PTB and different variables in terms of demographics, clinical symptoms, chest radiography, and laboratory test results. All variables evaluated in univariate analysis were then included in a multivariate regression model to identify potential predictors for PTB by calculating adjusted ORs with 95% confidence intervals (CI). We calculated the sensitivity, specificity, positive predictive value (PPV), negative predictive value (NPV), positive likelihood ratio (LR+), negative likelihood ratio (LR−), and area under the receiver operating characteristic (ROC) curve to evaluate the diagnostic performance of the common (and available) TB symptoms, CXR, TST, CD4+ cell level, and various combinations among them [18, 19]. All statistical analyses in this study were performed with STATA version 10.1 (StataCorp LP College Station, TX). A *P* value < 0.05 was considered statistical significant.

## 3. Results

### 3.1. Study Population

**Figure 1:**
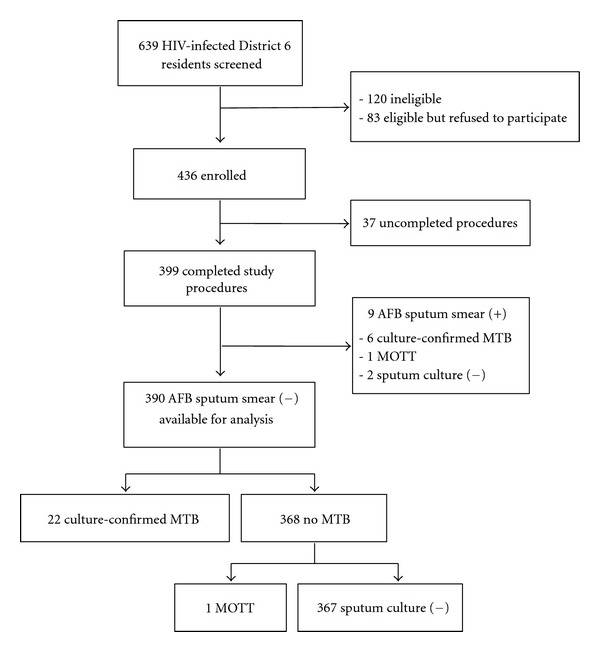
Patient enrollment flow chart. AFB-positive: having at least one AFB-positive sputum smear. AFB-negative: having both AFB-negative sputum smears.

A total of 639 HIV-infected patients who registered as District 6 residents and sought care at the An Hoa Clinic during the study timeframe were screened for eligibility by the study staff. There were 120 patients ineligible by the inclusion criteria and 83 patients eligible, but refused to participate. Most of the ineligible patients were currently receiving or had recently received (≤ 1 year) TB treatment. Among 436 HIV-patients eligible by the inclusion criteria who agreed to participate in the study, 37 patients were excluded due to incomplete procedures (6 withdrew before having the interview, 26 attended the interview then withdrew without having any test done, 4 missed CXR, and 1 missed sputum culture result). Of 399 HIV-infected patients completing all study procedures, 9 (2.3%) were sputum AFB smear-positive and were excluded. The data of 390 (97.7%) patients with sputum smear-negative results were analyzed further. Among these smear-negative patients, the frequency of sputum culture results positive for MTB, MOTT, and negative were 22 (5.6%), 1 (0.3), and 367 (94.1%), respectively (Figure 1). 

Of 390 AFB smear-negative HIV-infected patients, the majority were men (277/390 (71%, 95% CI 67–76%)) and a substantial proportion of the study sample was made up of injecting drug users (248/390 (63.6%, 95% CI 61.3–70.9%)). Among four common TB symptoms (cough, fever, weight loss, and night sweats, [20] cough and weight loss were reported by participants at the highest frequency, 25.9% (101/390, 95% CI 21.5–30.3%) and 20% (78/390, 95% CI 2.0–24.0%), respectively. One-fifth (21.5%) of participants had CD4+ cell level below 200/mm^3^ and one-third (31.3%) had chest radiograph classified as TB-CXR (Table 1). 

### 3.2. Correlation of Demographic, Clinical, and Laboratory Characteristics with Tuberculosis

 Our univariate analysis found that AFB smear-negative pulmonary TB was significantly associated with fever, chills, CD4+ cell level < 200/mm^3^, and TB-CXR. A multivariate regression model was developed from significant and clinically important covariates. The Hosmer-Lemeshow goodness-of-fit statistic for this model had a *P* value = 0.69, which indicated that the model fit well with the data [19]. This multivariate model had good discrimination with the area under the ROC curve (sensitivity versus 1 − specificity) of 0.83. Multivariate regression analysis found that only the CD4+ cell level below 200/mm^3^ and TB-CXR were significantly associated with AFB smear-negative PTB (*P* value < 0.01). Demographic characteristics, medical history, and common TB symptoms presented no significant association with AFB smear-negative PTB (Table 1). 

### 3.3. Performance of Symptom-Based Algorithm in Screening AFB Smear-Negative TB

Among 390 HIV-infected patients, a total of 143 patients were positively screened by the algorithm of four common TB symptoms (cough, fever, night sweat, and weight loss).  Table 2 presents the poor performance of different combinations of clinical predictors in screening AFB smear-negative individuals. The combination of four TB common symptoms could identify only 10/22 (46%, 95% CI 24.1–66.8%) TB cases with a positive likelihood ratio of 1.25 and a negative likelihood ratio of 0.85. Although the combination of symptoms and TST had 100% sensitivity (22/22 PTB cases identified), this algorithm was not a good predictor for ruling out PTB with a positive likelihood ratio of 1.32 and a specificity of 24%.

## 4. Discussion

Like other developing countries, the diagnosis of pulmonary tuberculosis in Vietnam relies mainly on clinical screening, CXR, and sputum smear examination. While recommended by the World Health Organization (WHO) for diagnosis of AFB smear-negative TB in HIV-infected patients, sputum culture is still not routinely available at the district-level HIV clinics in Vietnam [14]. Although some authors have suggested clinical symptoms can be significant predictors of smear-negative PTB, neither clinical symptoms nor TST were significantly associated with smear-negative PTB in our population [6]. Among available potential predictors, our data found only a decrease CD4+ cell level below 200/mm^3^ and an abnormal CXR (TB-CXR) as being significant predictors for PTB in HIV-infected patients having sputum AFB-negative smears.

Consistent with previous studies, our data failed to identify neither any single symptom nor combination of symptoms which could be a sensitive predictor for smear-negative, culture-confirmed PTB [6, 8]. Recently, the WHO has recommended using combination of cough, fever, weight loss, and night sweats as the initial screening tool to exclude active TB cases before initiating preventing treatment for persons living with HIV in most resource-constrained settings [20]. However, our data showed that symptom screening may miss up to 54% of AFB smear-negative PTB cases among HIV-infected patients (sensitivity = 46% and negative likelihood ratio = 0.85). We also found 12 (3%) new smear-negative PTB cases, which would be missed by the current routine diagnostic tools (Table 1). Therefore, results from this study confirm the need of routinely applying sputum culture for HIV-infected patients in Vietnam [21]. In public health settings where the sputum culture is not available, chest radiography in addition to the CD4+ cell level below 200/mm^3^ could be feasible but poor diagnostic tools for AFB smear-negative PTB in HIV-infected individuals. 

Among 22 AFB smear-negative PTB cases, 7 (32%) were classified as resistant to TB; of which, 5 cases were resistant to streptomycin, 1 case was resistant to streptomycin and isoniazid, and 1 MDR-TB case which was resistant to streptomycin, rifampin, isoniazid, and ethambutol (data not shown). Although this important issue still needs to be investigated further with a larger study, these findings strongly suggest the need for rapid and accurate diagnostic tools for detecting resistant TB cases in HIV patients having AFB-negative sputum smears. GeneXpert MTB/RIF, a rapid assay endorsed by the WHO [20], which can simultaneously identify MTB and its susceptibility to the most common first-line anti-TB drug—rifampin, would be a feasible choice and should be evaluated in the HIV-infected population in Vietnam. 

A similar study to our study in a setting of high TB prevalence suggested that smear-negative PTB cases were more likely to be confirmed by sputum culture as MOTT than in smear-positive PTB cases [6]. Our study found only two MOTT cases, which were equally distributed in the two groups of smear status-one in each. However, our sample size may not be powerful enough to evaluate the association between MOTT and AFB negative smear status. Further studies with larger sample sizes should be conducted to investigate thoroughly this important issue. As MOTT and MTB have distinct prognoses and therapy [22], a better understanding on their diagnostic characteristics would be useful in developing clinical algorithms for their differential diagnosis, especially in low-resource settings. 

Our study has some notable limitations. First, the study was conducted at a single clinic. However, as the majority (65%) of An Hoa Clinic's patients were District 6 residents, the clinic received most of the HIV-infected patients residing in the District [23], and the study subjects were consecutively enrolled from patients having AFB-negative sputum smears, our study sample should represent the district's smear-negative HIV-infected population. Second, our study used only one sputum culture for each patient as we followed the routine procedure often used in Vietnam. This single culture procedure may obviously underestimate the real number of PTB cases [24]. Third, the Löwenstein-Jensen sputum culture technique used in our study may miss some PTB cases [24]. Liquid-based culture technique such as the WHO approved MGIT should be deployed as a more rapid and accurate alternative for Löwenstein-Jensen technique where local resources allowed [24, 25]. The possible use of available nucleic acid amplification tests (NAATs) should also be considered to help enhance the detection of smear-negative tuberculosis [26]. 

In conclusion, diagnosis of smear-negative PTB in HIV-infected patients is a real challenge for clinicians at district-level HIV clinics in Vietnam where the sputum culture is not available. Our data showed the poor clinical performance of symptoms screening for AFB smear-negative PTB and confirmed the urgency of routinely applying sputum culture for HIV-infected individuals to improve the detection of not only smear-negative PTB but also TB disease in general. Where the sputum culture is not available, the algorithm of CXR plus CD4+ cell count could be a feasible but poor diagnostic tool. As no published data is available regarding MOTT in Vietnam, the issue of differentiation between MOTT and MTB should be further investigated. Future studies on more affordable, rapid, and accurate tests for TB infection would also be necessary to timely provide specific treatment for patients in need, reduce mortality, and minimize TB transmission to the general population.

## Figures and Tables

**Table 1:** Potential predictors for pulmonary tuberculosis in 390 HIV-infected persons having sputum smears negative for acid fast bacilli.

Characteristics	All patients	PTB	No PTB	Unadjusted OR	Adjusted OR^a^
	(*N* = 390)	(*n* = 22)	(*n *= 368)	(95% CI)	(95% CI)
Men	277 (71.0)	20 (90.9)	257 (69.8)	4.32 (0.99, 18.79)	6.82 (0.65, 72.24)
Median age, years (IQR)	30 (27–34)	31 (28–34)	30 (27–34)	0.98 (0.91, 1.06)	0.95 (0.86, 1.04)
Median household, person (IQR)	6 (4–7)	6 (5–8)	6 (4–7)	1.06 (0.94, 1.18)	1.08 (0.95, 1.23)
Incarcerated history	63 (16.2)	6 (27.3)	57 (15.5)	2.05 (0.76, 5.45)	0.91 (0.29, 2.90)
Smoker	308 (79.0)	20 (90.9)	288 (78.3)	2.78 (0.64, 12.14)	0.40 (0.03, 4.67)
Alcoholism	168 (43.1)	9 (40.9)	159 (43.2)	0.91 (0.38, 2.18)	0.78 (0.28, 2.18)
Intravenous drug users	248 (63.6)	17 (77.3)	231 (62.8)	2.02 (0.73, 5.59)	1.24 (0.32, 4.80)
TB treatment history	173 (44.4)	10 (45.5)	163 (44.3)	1.05 (0.44, 2.49)	0.89 (0.27, 2.92)
Receiving ART	225 (57.7)	11 (50.0)	214 (58.2)	0.72 (0.30, 1.70)	0.58 (0.18, 1.83)
Cough	101 (25.9)	6 (27.3)	95 (25.8)	1.08 (0.41, 2.83)	0.59 (0.16, 2.20)
Fever	24 (6.2)	4 (18.9)	20 (5.4)	3.87 (1.20, 12.50)*	3.33 (0.71, 15.66)
Weight loss	78 (20.0)	6 (27.3)	72 (19.6)	1.54 (0.58, 4.08)	1.61 (0.40, 6.44)
Sweats	11 (2.8)	1 (4.6)	10 (2.7)	1.71 (0.21, 13.95)	0.57 (0.04, 7.62)
Loss of appetite	38 (9.7)	4 (18.2)	34 (9.2)	2.18 (0.70, 6.82)	2.14 (0.47, 9.67)
Chills	34 (8.7)	5 (22.7)	29 (7.9)	3.44 (1.18, 9.99)*	1.84 (0.41, 8.19)
Fatigue	61 (15.6)	6 (27.3)	55 (14.9)	2.13 (0.80, 5.69)	0.74 (0.18, 3.05)
Positive TST	220 (56.4)	16 (72.7)	204 (55.4)	2.14 (0.82, 5.60)	2.36 (0.76, 7.33)
CD4+ cell/*μ*L <200	84 (21.5)	10 (45.5)	74 (20.1)	3.31 (1.38, 7.96)**	3.35 (1.20, 9.37)*
TB-CXR	122 (31.3)	14 (63.6)	108 (29.4)	4.21 (1.72, 10.33)**	4.38 (1.52, 12.61)**

^a^Adjusted in the multivariate model.

TB-CXR: abnormal radiograph suggestive of tuberculosis.

PTB: pulmonary tuberculosis due to *Mycobacterium tuberculosis.*

OR: odds ratio.

CI: confidence interval.

IQR: interquartile range.

*P value < 0.05.

**P value < 0.01.

**Table 2:** Performance of different algorithms in screening TB for 390 HIV-infected patients having sputum smear negative to AFB.

Combination of predictors	Sensitivity	Specificity	PPV	NPV	LR+	LR−	Area under ROC curve*
Combination of three clinical predictors							
Cough or fever or night sweats	8/22 (36)	266/368 (72)	8/102 (7)	266/280 (95)	1.31	0.88	0.54
Cough or night sweat or weight loss	9/22 (41)	236/368 (64)	9/141 (6)	236/249 (95)	1.14	0.92	0.52
Fever or night sweats or weight loss	8/22 (36)	285/368 (78)	8/91 (9)	285/299 (95)	1.61	0.82	0.57

Combination of four clinical predictors							
Cough or fever or night sweats or weight loss	10/22 (46)	235/368 (64)	10/143 (7)	235/247 (95)	1.25	0.85	0.55

Combination of four clinical predictors and TST							
Symptoms or TST	22/22 (100)	89/368 (24)	22/301 (7)	89/89 (100)	1.32	0	0.62

*ROC curve: receiver operating characteristic curve.

PPV: positive predictive value. NPV: negative predictive value.

LR+: positive likelihood ratio, calculated by sensitivity ÷ (1 − specificity).

LR−: negative likelihood ratio calculated by (1 − sensitivity) ÷ specificity [18].

Sensitivity, specificity, PPV, and NPV are reported as proportion (%).

CXR: chest radiography.

Cough: lasting ≥2 weeks in the past 4 weeks.

Fever: lasting ≥2 weeks in the past 4 weeks.

Night sweats: lasting ≥2 weeks in the past 4 weeks.

Weight loss: lasting ≥2 weeks in the past 4 weeks.
